# Route choices of transport bicyclists: a comparison of actually used and shortest routes

**DOI:** 10.1186/1479-5868-11-31

**Published:** 2014-03-06

**Authors:** Patricia Jasmin Krenn, Pekka Oja, Sylvia Titze

**Affiliations:** 1 Institute of Sport Science, University of Graz, Mozartgasse 14, 8010 Graz, Austria; 2 UKK Institute, Kaupinpuistonkatu 1, 33500 Tampere, Finland

**Keywords:** Bicycling, Environment, Bicycle lane, GIS, GPS

## Abstract

**Background:**

Despite evidence that environmental features are related to physical activity, the association between the built environment and bicycling for transportation remains a poorly investigated subject. The aim of the study was to improve our understanding of the environmental determinants of bicycling as a means of transportation in urban European settings by comparing the spatial differences between the routes actually used by bicyclists and the shortest possible routes.

**Methods:**

In the present study we examined differences in the currently used and the shortest possible bicycling routes, with respect to distance, type of street, and environmental characteristics, in the city of Graz, Austria. The objective measurement methods of a Global Positioning System (GPS) and a Geographic Information System (GIS) were used.

**Results:**

Bicycling routes actually used were significantly longer than the shortest possible routes. Furthermore, the following attributes were also significantly different between the used route compared to the shortest possible route: Bicyclists often used bicycle lanes and pathways, flat and green areas, and they rarely used main roads and crossings.

**Conclusion:**

The results of the study support our hypothesis that bicyclists prefer bicycle pathways and lanes instead of the shortest possible routes. This underlines the importance of a well-developed bicycling infrastructure in urban communities.

## Background

Physical inactivity continues to be a significant health problem in many developed countries. A growing body of evidence shows that bicycling is a potentially sustainable solution to improve public health [1]. According to the socio-ecological models of health, a multi-level approach is advocated for the improvement of physical activity behaviour [2].

The relationship between the built environment and the physical activity behaviour of a population has been given attention in the last few decades. Various studies have shown that urban environments can be correlated with physical activity [3-5]; however, studies focusing on the environmental correlates of bicycling as a means of transportation are scarce. Bicycle lanes, bicycle pathways, flat terrain, low traffic volume, and green and attractive areas have been shown to be correlated to cycling [5-8].

Two principal approaches have been used to investigate bicyclists’ routes in order to study the relationship between the environment and bicycling: questionnaires and global positioning systems (GPS). While questionnaires are subjective tools, GPS permits the objective measurement of routes [9,10]. GPS is a satellite-based global navigation system that can be used to identify an individual's location at any time and at any point on the earth’s surface. A further benefit of GPS is that it can be combined with geographic information systems (GIS) to analyse environmental features along the GPS route by the use of objective digital map data. The combined application of GPS and GIS is useful to identify environmental features that support or hinder bicycling in large study populations.

The actually used and shortest bicycling routes have been compared in a number of empirical studies in order to better understand the route choices of transport bicyclists. In most of these studies, the actual bicycling routes were assessed by the use of questionnaires [11,12] or the study participants were asked to trace their routes on a map [13,14]. To the best of our knowledge, actual bicycling routes have only been assessed objectively with GPS in a study undertaken by Winters and co-workers [15]. This study was conducted in Vancouver, Canada, and the results suggest the importance of the environmental characteristics such as bicycling facilities, connectivity, topography and land use. However, we lack empirical evidence of this type of study for Europe.

The aim of the present study was to improve our understanding of the environmental determinants of bicycling as a means of transportation in urban European settings by comparing the spatial differences between the routes actually used by bicyclists and the shortest possible routes. We examined differences with respect to distance, type of street, and environmental characteristics, assessed by GPS, self-reported bicycle trips and GIS.

## Methods

### Research setting and sample

The present study was conducted in the city of Graz in Austria, which has a population of nearly 300,000 and covers an area of approximately 127 km^2^. The city has a relatively large number of bicycle pathways, especially along the river Mur, which intersects the city from north to south. The modal share for transport bicycling is currently 14% [16]. The mild climate of Graz permits bicycling throughout almost the whole year. The bicycling infrastructure of Graz (120 km) consists of dedicated bicycle pathways, as well as bicycle lanes adjacent to streets. Furthermore, all side roads are subject to a speed limit of 30 km/h (= 19 miles/h) and are therefore quite attractive for bicycling. Traffic signals for bicyclists and pedestrians permit bicyclists to use designated bicycle pathways and to cross major roads safely.

The study participants were recruited from a previous study conducted in Graz, which is known as the "Bike-friendly city” study (n = 1000) [14]. In 2005, 80 study participants drew their most frequent bicycle trip on a map. In 2010, 70 members of the Graz study were asked to wear a GPS data logger for four days in order to record their daily trips. Forty-eight of the 70 participants used their bikes as a means of transportation, and were included in the analysis. Fifteen persons participated in both bicycling studies. Thus, data concerning 113 participants (80 − 15 + 48) were available for analysis. The demographic data of all the study participants were derived from the “Bike-friendly city” study [14]. The current study was approved by the ethics committee of the Medical University of Graz (#21-349).

### Actually used bicycling routes

The 80 single bicycle trips from the "Bike-friendly city" survey were transferred from analogue maps to a GIS. The trips were digitized using ArcGIS 9.1, based on the street centreline network data set. Bicycle trips from the GPS study (several different trips from one person) were smoothed using MATLAB and then imported into the GIS. Smoothing was undertaken automatically by eliminating single GPS data points with unfeasible speed or acceleration. The street network only consisted of single lines (no width), while the GPS data included the width of streets when they are crossed. Therefore, the GPS trips were generalized to straighter lines using the GIS functions to ensure comparability with the street network with respect to distances. Redundant trips were eliminated from the data set. Finally, both data sets were combined to yield 278 different bicycle trips, while each of the 113 participants was weighted equally for environmental analysis along the routes. In other words, participants who undertook only one bicycle trip were considered as equal to those who undertook more trips.

### Shortest routes

The street network and the bicycle paths in the city were used to calculate the shortest distance between origin and destination. Information regarding starting and ending points permitted calculation of the shortest possible routes, with no restriction applied for speed limits, topography, one-way streets or turning restrictions. Shortest routes were generated using the Network Analyst tool included in ArcGIS 9.1. By comparing the actual and shortest possible routes, detours were calculated absolutely in metres as well as relatively in percentages.

### Preparation of geographic data

A number of geographic data maps were prepared to identify the environmental characteristics that were correlated to bicycling as a means of transportation. The environmental characteristics that were correlated to bicycling in other studies were included primarily to examine the results for Graz.

Maps were acquired from administrative institutions and the OpenStreetMap portal. The following thematic maps were extracted from the data sources and used for GIS analysis along the routes: street types (bicycle pathways, bicycle lanes, side streets without parallel bicycle lanes, main roads without parallel bicycle lanes), traffic lights, crossings, green areas, urban trees, aquatic areas, topography, residential zones, industrial zones, population density, land-use mix, and shops and services. Quantum GIS 1.6. and ArcGIS 9.1. were used for the spatial analysis. A street network, including all types of streets, bicycle paths and lanes, was generated in order to calculate street-based distances. Each street type was abstracted to its centreline of the street.

To derive the built environmental characteristics along the route, a buffer distance of 25 m was used to define the immediate neighbourhood of the route. This definition resulted from numerous tests performed in the city of Graz, and has been suggested in a similar study [17]. Different types of GIS methods were used within the route buffer employing ArcGIS 9.1.

For each street type, we calculated the number of metres and then determined the proportion of the street type along the total trip length. For point data such as crossings, the number of points were quantified and scaled as points per 1 km. For polygon data, the percentage of specific types of areas within the route buffer was calculated. Topography along the route was assessed by summing up the gradient class values and scaling them per kilometre. The sum of class values yielded more feasible data than those derived from mean topography values along the routes. The land-use mix was calculated according to the definition proposed by Frank et al. [18]. We included four types of land use: residential areas, industrial areas, traffic areas, and green and aquatic areas. The index ranges from 0 to 1; 0 stands for only one land-use type, while 1 indicates a well-distributed land-use area. The density of the population in Graz is divided into 125-m cells on official maps. The number of citizens en route was calculated proportionately for the buffer areas that intersect with the cells, and the results were scaled per hectare.

### Statistical analysis

The statistical analysis was performed using SPSS, version 19. The normal distribution of each variable was tested using the Kolmogorov-Smirnov test. To calculate the differences in distance, street type and environmental characteristics between the actual and shortest possible routes, paired t-tests were used for normally distributed variables and Wilcoxon tests for the skewed variables.

## Results

### Demographic data

The demographic characteristics of the study sample are shown in Table 1. More women than men participated in the study (55% vs. 45%), 40% were younger than 35 years, and education levels were equally distributed in the groups.

**Table 1:** Descriptive characteristics of the study sample (n = 113)

	**N**	**%**
**Gender**		
Female	62	55
Male	51	45
**Age**		
<35 years	45	40
35–50 years	45	40
>50 years	23	29
**Education**		
Compulsory school	18	16
Apprenticeship, intermediate vocational degree	30	27
High-school diploma	32	28
University graduates	33	29
**Body mass index**		
<25	92	81
≥25	21	19

The study sample, which only consisted of bicyclists, was compared with the representative sample of the original "Bike-friendly city" study (n = 1000), which represented the general population. The participants of the present study had a significantly higher level of education (university degree) (p = 0.001) compared to the representative sample (29% vs. 17%). Moreover, significantly fewer people were overweight in the present study, as assessed by their BMI (19% vs. 30%, p = 0.014). No statistical differences were found between the two samples with respect to gender or age (p > 0.05).

### Distances

The 278 bicycle trips were not normally distributed. The median length of the bicycle trips was 2.3 km; 78% of all trips were shorter than 4 km. The mean difference between the actual trips and shortest possible routes was 277 m (median, 168 m). About two-thirds of the bicyclists (63%) detoured more than 100 m, and 86% detoured more than 5 m in comparison to the shortest possible route. The percentage of the detours ranged between 0 and 37%. About 14% of the actual trips did not differ from the shortest possible routes. Table 2 provides an overview of the actually used and the shortest possible routes. The minimum difference in distance was zero for trips without a detour. The Wilcoxon test revealed a significant difference between the actually used and the shortest possible routes (Z = −14, 256; p < 0.001).

**Table 2:** Differences in distance between the actually used and the shortest possible bicycling routes (n = 278)

	**Actual trips (m)**	**Shortest trips (m)**	**Detour (m)**	**Detour (%)**	**Significance**
Median	2337	2146	168	7.6	<0.001
IQR	2113	1860	343	10.1	
Minimum	383	377	0	0	
Maximum	13864	12494	1946	37	

IQR: Inter-quartile range.

Comparisons between men and women did not reveal any significant differences with regard to trip distances or detours. No significant difference was registered between the bivariate variables of age (≤45, >45 years), education (<high-school diploma, ≥high-school diploma), BMI (<25, ≥25), trip distances and detours.

### Environmental characteristics of bicycling routes

The differences between the actually used and the shortest routes showed that actual trips with almost no detours did not necessarily follow the same course as did the shortest trips. The results of the GIS analyses are shown in Table 3, divided into the categories of traffic, green/aesthetics, topography, and other land-use types. The significant differences are highlighted in bold.

**Table 3:** Comparison of the environment along the actual and shortest possible routes (n = 113)

**Variable**	**Mean actual route (SD)**	**Mean shortest route (SD)**	**Difference (actual − shortest)**	**Significance**
**Traffic**				
Bicycle pathways^a^	30.0 (±28,3)	19.3 (±20,8)	10.7	**<0.001**
Bicycle lanes^a^	31.3 (±26.7)	30.6 (±22.7)	0.5	0.723
Side roads without bicycle lanes^a^	21.6 (±14,5)	24.6 (±14,6)	−3.0	**0.001**
Main roads without bicycle lanes^a^	9.8 (±17.7)	21.2 (±28.1)	−10.4	**<0.001**
Traffic lights^b^	1.5 (±1,1)	1.7 (±1,0)	−0.2	**0.001**
Crossings^b^	1.3 (±0.9)	1.6 (±1.0)	−0.3	**<0.001**
**Greenery/aesthetics**				
Green and aquatic areas^c^	19.2 (±10.9)	14.5 (±7.7)	4.7	**<0.001**
-Urban trees^b^	44.9 (±30.7)	42.2 (±27.3)	2.7	0.089
-Sports and recreation areas^c^	7.7 (±8.7)	4.3 (±5.2)	3.4	**<0.001**
-Playing fields^c^	0.9 (±1.2)	0.8 (±1.5)	0.1	**0.042**
-Forests^c^	0.6 (±1.8)	0.5 (±1.5)	0.1	0.341
-Aquatic areas^c^	2.9 (±4.6)	1.3 (±2.1)	1.6	**<0.001**
**Topography**				
Steepness^d^	0.2 (±0.6)	0.4 (±0.9)	0.2	**0.031**
**Other land use**				
Residential areas^c^	28.6 (±20.1)	28.1 (±19.4)	0.5	0.778
Industrial and commercial areas^c^	3.2 (±4.7)	3.2 (±4.7)	0.0	0.870
Residential density^e^	64.2 (±30.2)	67.6 (±29.2)	−3.4	**0.034**
Land-use mix^f^	0.82 (±0.1)	0.79 (±0.1)	0.02	**<0.001**
Shops and services^b^	4.4 (±4.5)	5.4 (±5.0)	−1.0	**<0.001**

a = % of the trip length, b = points per 1 km trip length, c = % of the environment along the trip (15 m buffer), d = sum of the gradient values per 1 km trip (0: flat – 8: very steep), e = residences per ha in the route neighbourhood, f = value from the LUM formula (Frank et al., [18]), SD = standard deviation.

* = paired t-tests for normally distributed data and Wilcoxon tests for data with a skewed distribution.

The traffic data showed that bicyclists used the existing bicycling infrastructure. Nearly two-thirds of the actual bicycle trips (30.0% on bicycle pathways and 31.3% on bicycle lanes) were conducted on bicycle lanes or paths. Bicycle paths were used significantly more often on actual routes than by those using the shortest routes. Marked and significant differences were registered for main roads without bicycle lanes, and it was found that bicyclists avoided these busy streets (9.8% of these main roads were on the actual route vs. 21.2% on the shortest possible route). The sum of the proportions of the different street types did not result in 100% because gateways to houses and very narrow laneways were not represented in GIS street data. Actually used routes had fewer traffic lights and fewer crossings than the shortest possible routes.

On average, the actual bicycle trips were conducted in significantly more green and aquatic areas than the shortest trips. A comparison of sports and recreation areas, playing fields and aquatic areas revealed (highly) significant data (positive as regards bicycling). The results indicate that bicyclists select routes with more green and aquatic areas.

A significant difference in topography was noted between the actual and the shortest routes. It was found that bicyclists make detours and opt for routes that are less hilly than the shortest routes.

No significant differences between the two types of routes were observed with respect to residential, industrial and commercial areas. However, residential density and the number of shops and services were significantly higher along the shortest possible routes. The mixture of land-use types, calculated as the land-use mix value, was significantly higher along actual bicycling routes than along the shortest routes.

## Discussion

The present study, conducted in the city of Graz, Austria, showed differences in distance and environmental characteristics between the actually used and the shortest possible bicycling routes. The results indicate that bicyclists make detours in order to use bicycle pathways and flat and green areas and to avoid traffic lights and crossings.

### Distances

The mean difference in distance was 277 m (median, 168 m), whereas two-thirds of the actual routes differed by a maximum of 10% from the shortest distances. On average, in Graz, the actual distances were 7.6% longer than the shortest possible ones. A study in Vancouver, Canada, comprising 50 bicycle trips yielded similar data [15]. Winters and co-workers found that the actual bicycling routes were on average 8.3% longer than the shortest possible routes. In a study conducted in Phoenix, USA, Howard and Burns [11] investigated 150 regular bicyclists and found that bicycle trips were on average 10% longer than the shortest possible routes from origin to destination. In a Japanese study comprising 754 bicyclists, the detour ratios of bicyclists ranged between 6 and 16% [12]. Aultman-Hall and co-workers [13] analysed the trips of 79 bicyclists in Guelph, Canada, and found that actual bicycling routes were on average 11% longer than the shortest possible routes. Detours were much fewer in Graz than they were in these international studies. We now believe that the short detours in the present study were due to the compactness of the city of Graz, which is also typical for other European cities, and which is less true for cities in Australia or America/Canada. In European cities, everything is close by, and the median trip length was also lower than in, for example, Canadian cites (2.3 km in Graz vs. 3.7 km in Vancouver). Moreover, European cities like Graz have well-developed bicycling infrastructure, and so the need for detours may be less compared to cities with poorer bicycling facilities.

### Environmental characteristics

We observed differences in environmental characteristics between the actually bicycled and the shortest possible routes. Bicyclists used many bicycle pathways and lanes as well as side streets, whereas they rarely used main roads without bicycle lanes. The percentage of bicycle paths and lanes along the actual routes was more than 61%, but was only about 50% on the shortest possible bicycling routes. Studies in other countries yielded similar results. Bicyclists in Vancouver had about 50% bicycle pathways on their bicycling routes, whereas only 21% of bicycle pathways were on the shortest possible route [15]. In the study in Phoenix, 51% of bicycle trips were conducted on the bicycling infrastructure, whereas only 39% were conducted on the shortest possible routes [11].

Dill (2009) reported that 49% of trips made by regular bicyclists were conducted on bicycle pathways or lanes in Portland, although these lanes account for a mere 8% of the city street network [19]. According to Krizek et al. (2007), bicyclists in Minneapolis used routes that were 68% longer than the shortest routes in the bicycling infrastructure [20]. These data make it clear that bicyclists prefer bike-friendly routes to shorter ones.

Main roads and other roads with high-speed motorized transport were less used for bicycling in Graz, so we identified them as bike-unfriendly. In this regard, our data concurred with those reported in other studies (Int-Panis et al., [21]; Winters and Teschke, [8]; Winters et al., [15]). However, Aultmann-Hall et al. [13] found that bicyclists used more main roads than bicycle pathways. This inconsistency may be explained by the differences in street networks, the availability of bicycling infrastructure, and the connectivity in cities. Graz is known to be a bike-friendly city and offers a large number of bicycle pathways and lanes compared to other cities. Therefore, the bicyclists’ trips consisted on average of 61.3% dedicated bicycle pathways and lanes. Although side roads in Graz have speed limits of 30 km/h and are therefore quite comfortable for bicycling, bicyclists were found to use dedicated bicycle pathways and lanes. The comparison of the shortest and actually used bicycling routes revealed a greater percentage of side roads for the shortest possible routes (21.2% vs. 9.8%). Owing to the small number of bicycle paths and lanes along the shortest routes, a large number of these paths and lanes were obviously located along side roads. Therefore, data concerning side roads have to be interpreted in relation to other types of roads. It was found that bicyclists use detours to avoid traffic lights and crossings, and thus save time. This supports the theory of Dill (2009), who mentioned that bicyclists in Portland give importance to reducing their waiting time due to stop signs/lights [19].

Significant differences were registered between the actual and shortest routes with regard to the combined variable of green and aquatic areas, and it was found that bicyclists preferred routes along the green and aquatic areas. This is in accordance with data reported by Titze and co-workers (2010), who found that an attractive neighbourhood with a large number of trees is positively correlated with bicycling behaviour in Austria [22].

Topography played a role in selecting bicycling routes: the actual routes were significantly flatter than the shortest possible routes. Dill et al. [19] and Aultman-Hall et al. [13] found that bicyclists avoid steep areas. Int-Panis and co-workers (2010) observed that the number of bicyclists in Belgium was dependent on topography, and that a flat terrain encourages commuter bicycling [21]. In Vancouver, no difference in topography was observed between the actual and shortest trips; the authors claim that the topography in the study area was too similar to show major differences [15].

The condition that a high land-use mix (LUM) is regarded as bike-friendly was confirmed. The land-use mix was higher along the actually used routes than along the shortest routes. In Portland, this was one of the reasons why the mix of land uses was supported by policy-makers [19]. However, the results for the land-use mix depend on the way the land-use mix is defined. In the present study, the land-use mix was calculated according to the definition proposed by Frank et al. [18]. We included four types of land use: residential areas, industrial areas, circulation areas, and green and aquatic areas. The index ranges from 0 to 1; 0 stands for only one land-use type, while 1 indicates a well-distributed land-use area.

The numbers of shops and services showed a significant negative relationship with bicycling behaviour, and it was found that bicyclists avoided areas with large numbers of shops and restaurants. Firstly, this seems to contradict the results of the land-use mix. However, according to the definition above, restaurants and shops belong to the same land-use type, and so these shopping areas do not have land-use diversity. From the results, we conclude that bicyclists who use their bikes as a means of transportation select rapid routes without stops in order to save time and maintain their speed. The same was observed in Ontario, where bicyclists avoided the downtown area with busy streets and large numbers of pedestrians [13]. Thus, bicyclists use different criteria to select their routes than do pedestrians. Pedestrians give priority to facilities such as shops and services, as well as good connectivity [23,24]. Nevertheless, we believe that bicycling infrastructure should be in the vicinity of shops and services to permit intermittent shopping stops for bicyclists.

The strength of the present study was that we used objective measurement instruments to identify the environment along bicycling routes. Based on existing digital map data, similar analyses could be performed for different regions. Another strength was that more than 40% of the trips were recorded objectively using GPS devices. As regards the limitations of the study, it should be mentioned that we combined GPS-recorded trips and self-reported ones. Therefore, all the trips may not have been conducted on the actual courses, and the self-reported trips may have been biased. We could not verify whether the participants actually did use the specified route. As this was a cross-sectional study, the results show correlations between environmental characteristics and bicycling behaviour. Evaluation of the influence of the environment will require experimental study designs.

Although our sample size was relatively small, we expect that the results can be generalized for similar European cities. Further work needs to be undertaken in other European cities to confirm the validity and reliability of the results. Furthermore, the present study provides some ideas of which environmental characteristics support or hinder bicycling for transportation. Given that the characteristics of bicyclists and non-bicyclists differ, efforts to increase uptake of bicycling for transport among non-cyclists will require broad-ranging interventions that target personal and social correlates as well as environmental factors [25]. Based on the study results, the next step will be to map and afterwards to identify city regions in Graz that are bike-friendly or bike-unfriendly according to the correlation results. Since GIS typically does not provide data on frequently changing characteristics on the micro-scale (eg. advertising, rubbish and street conditions), it would also be interesting to make a similar study using digital cameras to assess the environment. The present results could then be extended to correlates of the micro-environment, such as private fences, billboards or dirty streets.

## Conclusions

The aim of the study was to investigate whether the built environment differs along actually used bicycling routes as compared to the shortest possible routes. The data showed that bicyclists used bicycle pathways/lanes, flat roads, and attractive areas rather than the shortest possible routes. Based on the study results and further GIS analysis, it will be possible to visualize the urban bicycling environment. Finally, suggestions could be made to policy-makers and city planners as to where the bicycling conditions should be further improved.

## Competing interests

The authors declare that they have no competing interests.

## Authors’ contributions

PK conducted the study, executed the GIS analysis and prepared the manuscript. ST made substantial contributions to the conception and design of the study, the analysis and interpretation of data and the drafting of the manuscript. PO was involved in the conception and interpretation of the study results and revised the manuscript for important intellectual content. All authors read and approved the final manuscript.
